# Clinical features, immunologic parameter and treatment outcome of Chinese tuberculosis patients with or without DM

**DOI:** 10.3389/fmed.2024.1386124

**Published:** 2024-06-12

**Authors:** Fengjun Tong, Jie Lai, Zhenhui Lu, Zhijian Bao, Junyan Cao

**Affiliations:** ^1^Department of Infectious Diseases, Qingchun Hospital, Hangzhou, China; ^2^Zhejiang Tuberculosis Diagnosis and Treatment Center, Hangzhou Red Cross Hospital, Hangzhou, China

**Keywords:** pulmonary tuberculosis, diabetes, cytokines, lymphocyte subpopulation, treatment response

## Abstract

**Background:**

The coexistence of diabetes mellitus (DM) and pulmonary tuberculosis (PTB) poses a significant health concern globally, with their convergence presenting a considerable challenge to healthcare systems. Previous research has highlighted that comorbidities can mutually influence and exacerbate immune disorders. However, there is a paucity of data on the impact of DM on immunological features and treatment responses in the TB population in China.

**Methods:**

From January 2020 to June 2022, 264 cases of pulmonary tuberculosis patients (82 DM patients and 182 non-DM patients) hospitalized in our center were selected. 80 patients with TB with DM (TB-DM) and 80 patients with TB without DM (TB-NDM) were enrolled into the final analysis by propensity score matching for age, gender and involved lung field at a ratio of 1:1. The clinical characteristics, immunological features and treatment response were compared between the two groups.

**Results:**

After propensity score matching, no differences in the general features such as age gender, involved lung field, the incidence of retreatment and WBC count were found between the two groups. Compared to TB-NDM group, the TB-DM group exhibited a higher positive rate of sputum smear and incidence of cavitary lesions. Immunological features analysis revealed that the TB-DM patients had higher levels of TNF-α [pg/ml; 8.56 (7.08–13.35) vs. 7.64 (6.38–10.14) *p* = 0.033] and IL-8 [pg/ml; 25.85 (11.63–58.40) vs. 17.56 (6.44–39.08) *p* = 0.003] but lower CD8+ T lymphocyte count [cells/mm3; 334.02 (249.35–420.71) *VS* 380.95 (291.73–471.25) *p* = 0.038]. However, there was no significant difference in serum IL-6 concentration and CD4+ T lymphocyte count between the two groups. After 2 months of anti-tuberculosis treatment, 39 (24.4%) cases had suboptimal treatment response, including 23 (28.7%) TB-DM patients and 16 (20%) TB-NDM patients. There was no difference in suboptimal response rate (SRR) was found between the two groups (*p* = 0.269). The multivariate logistic regression analysis indicated that retreatment for TB [AOR: 5.68 (95%CI: 2.01–16.08), *p* = 0.001], sputum smear positivity [AOR: 8.01 (95%CI: 2.62–24.50), *p* = 0.001] were associated with SRR in all participants, and in TB-DM group, only sputum smear positivity [AOR: 16.47 (1.75–155.12), *p* = 0.014] was positive with SRR.

**Conclusion:**

DM is a risk factor for pulmonary cavity formation and sputum smear positivity in TB population. Additionally, TB-DM patients is characterized by enhanced cytokine responses and decreased CD8+ T lymphocytes. The retreatment for TB and sputum smear positivity were associated with the occurrence of suboptimal treatment response.

## Introduction

Tuberculosis (TB) is a chronic infectious disease caused by *Mycobacterium tuberculosis* (MTB) and mostly manifests as a pulmonary disease, but can also affect extra-pulmonary sites (1). Although increasing countries around the world have performed several endeavors to prevent and control TB, it remains one of the most common causes of death from a single infectious pathogen (2). In addition, the prevalence of diabetes mellitus (DM) has progressively increased worldwide in recent years. There are more than 100 million diabetic patients, and the prevalence of diabetes in China is about 11.6%, ranking first in the world (3). Notably, the incidence of tuberculosis infection appears to be higher in DM patients (4).

Studies revealed that the occurrence and development of TB were closely related to the level of the body’s immune response. Both intrinsic and acquired immune functions are impaired in DM patients, promoting primary infection or reactivation of MTB, thereby increasing the incidence of TB (5). On the other hand, the presence of DM also appears to be related to heightened severity of TB disease among the infected individuals and to exert detrimental impacts on both disease manifestation and clinical outcomes (6). However, the controversy remains, as studies have shown that DM was not associated with more severe TB (6, 7).

It was reported that DM was associated with impaired immune response of innate immune cells, such as macrophages, dendritic cells (DCs), and natural killer (NK) cells in patients with TB (8). In addition, the lymphocyte subset function and phenotypic differentiation were also affected by the DM (9). Kumar et al. found that compared to individuals with TB without DM (TB-NDM), those TB with DM (TB-DM) patients were characterized by enhanced cytokine response including higher interferon-gamma (IFN-γ), tumor necrosis factor-α (TNF-α), and interleukin-6 (IL-6) indicating chronic inflammation underlying diabetes potentially contributes to increased immune pathology and poor control of tuberculosis disease (10). Exploring the different immune characteristics and treatment responses between TB-DM and TB-NDM patients helps understand the impact of DM on TB and even helps to improve disease management.

Currently, the data on Chinese TB-DM patients are uncomprehensive. Several studies illustrated well the basic clinical characteristics and immunological features of Chinese patients with TB-DM based on a small Chinese population (11, 12). Therefore, further studies are necessary to enrich the data of TB-DM patients in China. To better understand the role of DM in TB patients, we conducted a study. In this retrospective study, propensity score matching was conducted to minimize the potential selection biases. We compared the differences in the lymphocyte subset, levels of inflammatory cytokines such as IL-6, IL-8, and TNF-α and treatment response between TB-DM and TB-NDM groups, aiming to provide an overview of clinical characteristics, outcomes of TB between Chinese DM and non-DM patients.

## Method

### Study design and participants

This is a retrospective, observational clinical study conducted at Qingchun Hospital, Zhejiang province. Between January 2020 and July 2022, 284 TB patients (82 TB-DM and 182 TB-NDM) were retrospectively enrolled into this study. Pulmonary TB was diagnosed according to the standard WS288-2017 Tuberculosis Diagnosis (13). The diagnosis of diabetes mellitus was in line with guidelines for the prevention and treatment of type 2 diabetes in China (2020 edition), as fasting blood glucose ≥7 mmol/L, glycosylated hemoglobin was ≥6.5%, self-reported diabetes diagnosed by a doctor, or taking treatment of antidiabetic medications (14). And uncontrolled-DM was defined as a plasma glycated hemoglobin A1c (HbA1c) level ≥ 7.0%. Participants whose BMI **≥24 kg/m**^**2**^ were regarded as overweight. All patients were on first-line anti-tuberculosis treatment at least 2 months before the interview date. Patients suffering severe somatic disorders such as heart failure, chronic obstructive pulmonary disease (COPD), liver cirrhosis, chronic kidney disease, neoplasm or immunological deficiency were excluded. Subjects with a previous history of resistance were also excluded, as were pregnant or breastfeeding women.

### Procedure

**Figure 1:**
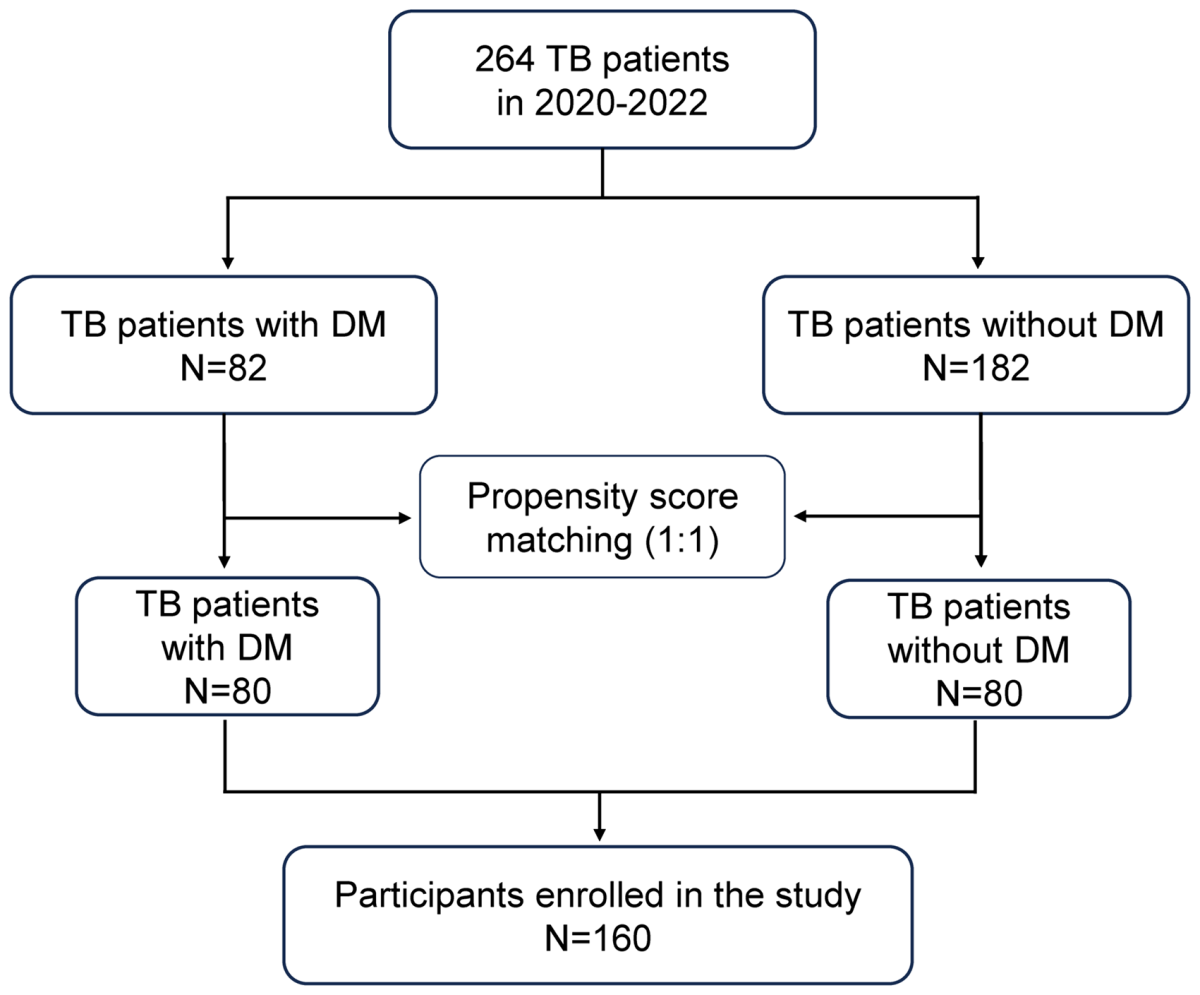
Study flowchart for patient selection. TB, tuberculosis; DM, diabetes mellitus.

Demographics, clinical presentations, laboratory test results, and imaging findings were retrospectively collected from the electronic medical records system of our center. Once PTB was diagnosed, all patients were treated with a standard four-drug combination anti-tuberculosis treatment with Rifampicin, Isoniazid, Pyrazinamide, and Ethambutol according to the guideline (13). Follow-up data of patients were collected 2 months after standard anti-tuberculosis therapy. The criteria for significant absorption of lung lesions involve a ≥ 50% decrease in the volume of measurable lesions or lung cavities becoming close. In contrast, the criteria for absorption of lung lesions entail a ≤ 50% decrease in the volume of measurable lesions or lung cavities diminishing in size but not becoming close. No absorption: no change in lung lesions or lung cavities and even enlarged. For patients with negative sputum smear who meet the criteria for absorption of lung lesions after 2 months of therapy, their condition is considered improved Patients whose pulmonary lesions had not been absorbed or whose sputum smear remained positive after treatment were regarded as suboptimal treatment response. Using the propensity score, we conducted propensity score matching for age, gender, and involved lung field to create 1:1 matched sets of TB patients with DM and without DM controls at a matching tolerance of 0.02. The flowchart for patient selection is shown in Figure 1.

### Immunological assays

Peripheral blood samples were collected after a minimum of 8 h of fasting and placed in heparin anticoagulant tubes. Plasma was harvested by centrifuging blood at 3000 rpm for 10 min. Then the magnetic particle-based chemiluminescence immunoassay was used to measure the levels of IL-6, IL-8, TNF-α according to the instructions of the cytokine kit (Sharay Bio-Tech, China). CD4+ T lymphocytes and CD8+ T lymphocytes counts were performed using fresh whole blood by FACS Calibur flow cytometry (Becton–Dickinson, United States).

### Statistical analysis

The data were entered in Microsoft Excel (2016) and analyzed in SPSS 25.0 software (IBM SPSS Statistics 25.0). GraphPad Prism 9.0 software (Prism, CA, USA) was employed for generating original images. Continuous variables are presented as the median [interquartile range, (IQR)]. Categorical variables are represented as numbers (percentage). To compare continuous variables, the Mann–Whitney U test was used. Chi-square or Fisher’s exact test was utilized to compare categorical variables. Binary logistic regression analysis to performed to determine crude odds ratios (OR) for factors associated with suboptimal treatment response. To ensure that important variables were not overlooked during the initial screening process, factors with significance (P<0.100) in univariable logistic regression analysis were included in the multivariate model and the adjusted odds ratios (AOR) along with their 95% confidence intervals (CI) were determined. In the final analysis, a two-tailed *p*-value less than 0.05 was considered significant.

### Ethics approval

The research protocols were conducted in compliance with the 1975 Declaration of Helsinki and obtained ethical approval from the Ethics Committee of the Qingchun Hospital, Zhejiang province (Approval Number: 2024-002-LW). All data were analyzed anonymously. The committee waived the necessity for obtaining written informed consent from the participants. This study was registered with ChiCTR.org.cn (ChiCTR2400080699).

## Results

### Baseline characteristics of participants

After propensity score matching, 160 patients were finally enrolled in this study. The general features of the patients included in the study are synthesized in Table 1. It was found that the median age of the participants was 47 (IQR: 39–51), the male was the dominant gender (93.1%), the median number of involved lung fields was 3 (IQR: 2–5). There were no statistically significant differences in age, gender, presenting WBC count, and involved lung field between the two groups. However, compared to TB-NDM group, TB-DM patients weighed more and had a higher proportion of overweight (22.6% vs. 7.5% *p* = 0.008). In addition, a higher positive rate of sputum smear was observed in TB-DM group (53.8% vs. 33.8%, *p* = 0.017). The incidence of cavitary lesions was also obviously higher with TB-DM compared with placebo (56.3% vs. 38.8% *p* = 0.039). In addition, patients with TB-DM had greater levels of C reactive protein (CRP) (*p* = 0.002) and erythrocyte sedimentation (ESR) (*p* = 0.001) on admission, indicating a more severe inflammatory response in TB-DM patients. No difference in the incidence of retreatment for TB between groups was observed. The detailed characteristics before and after propensity score matching were shown in Supplementary Table S1.

**Table 1:** The demographic characteristics of TB patients with and without DM.

**Parameter**	**All (*N* = 160)**	**TB-DM (*N* = 80)**	**TB-NDM (*N* = 80)**	***P* Value**
**Age [year (IQR)]**	47 (39–51)	47 (39–50)	46 (39–53)	0.737
**Gender [%(*n*)]**				
Male	149 (93.1)	73 (91.3)	76 (95.0)	0.534
**BMI [kg/m2 (IQR)]**	19.85 (21.24–23.04)	22.00 (19.68–23.71)	20.85 (19.88–22.28)	0.095
**Overweight [*n* (%)]**	24 (15.0)	18 (22.6)	6 (7.5)	**0.008**
**Involved lung fields [n (IQR)]**	3 (2–5)	3 (2–5)	3 (2–4)	0.818
**Involved lung fields ≥ 3 [*n* (%)]**	68 (42.5)	33 (41.3)	35 (43.8)	0.873
**Retreatment for TB [*n* (%)]**	51 (31.9)	26 (32.5)	25 (31.3)	0.999
**Sputum smear positivity [*n* (%)]**	70 (43.8)	43 (53.8)	27 (33.8)	**0.017**
**Lung cavitary lesions [*n* (%)]**	76 (47.5)	45 (56.3)	31 (38.8)	**0.039**
**Blood test**				
WBC [×109/L (IQR)]	6.00 (4.90–7.08)	6.20 (5.03–7.48)	5.70 (4.80–6.78)	0.110
CRP [mg/L (IQR)]	5.65 (2.00–31.65)	11.30 (2.28–45.73)	4.00 (1.60–18.08)	**0.002**
ESR [mm/h (IQR)]	16.00 (7.25–27.75)	18.56 (10.00–46.63)	12.00 (6.00–20.23)	**0.001**
**Anti-hyperglycemic therapy**				
Oral hypoglycemic agents	40 (50.0)	40 (50.0)	–	–
Insulin	39 (48.8)	39 (48.8)	–	–
Combination therapy	1 (1.2)	1 (1.2)	–	–
**HbA1c**				
≥7.0% [*n* %]	61 (76.3)	61 (76.3)	–	–
<7.0% [*n* %]	19 (23.7)	19 (23.7)	–	–

TB, tuberculosis; DM, diabetes mellitus; NDM, non-diabetes mellitus; WBC, white blood cell count; CRP, C reactive protein; ESR, erythrocyte sedimentation rate; HbA1c, glycated hemoglobin A1c; IQR, interquartile range. *P* values were calculated by Mann–Whitney test (continuous variables) or Chi-square test (categorical variables). P values in boldface type indicate significance at a P value of <0.05.

For the patients with TB-DM, 40 (50%) were getting oral hypoglycemic agents (OHA), 39 (48.8%) were on insulin whereas 1 had an OHA plus insulin prescription. Furthermore, 61 (76.3%) patients had poor glycemic control with HbA1c level ≥ 7.0%. To investigate the influence of BMI and diabetes control status on the severity of tuberculosis, we conducted a comparative analysis of the relevant measures from patient in TB-DM group. It was observed that neither the number of involved lung fields nor the incidence of lung cavitary lesions or sputum smear positivity were associated with being overweight. Additionally, the above parameters of tuberculosis severity were not found to be statistically relevant with poor glycemic control.

### The impact of diabetes on immunological responses in TB patients

**Figure 2:**
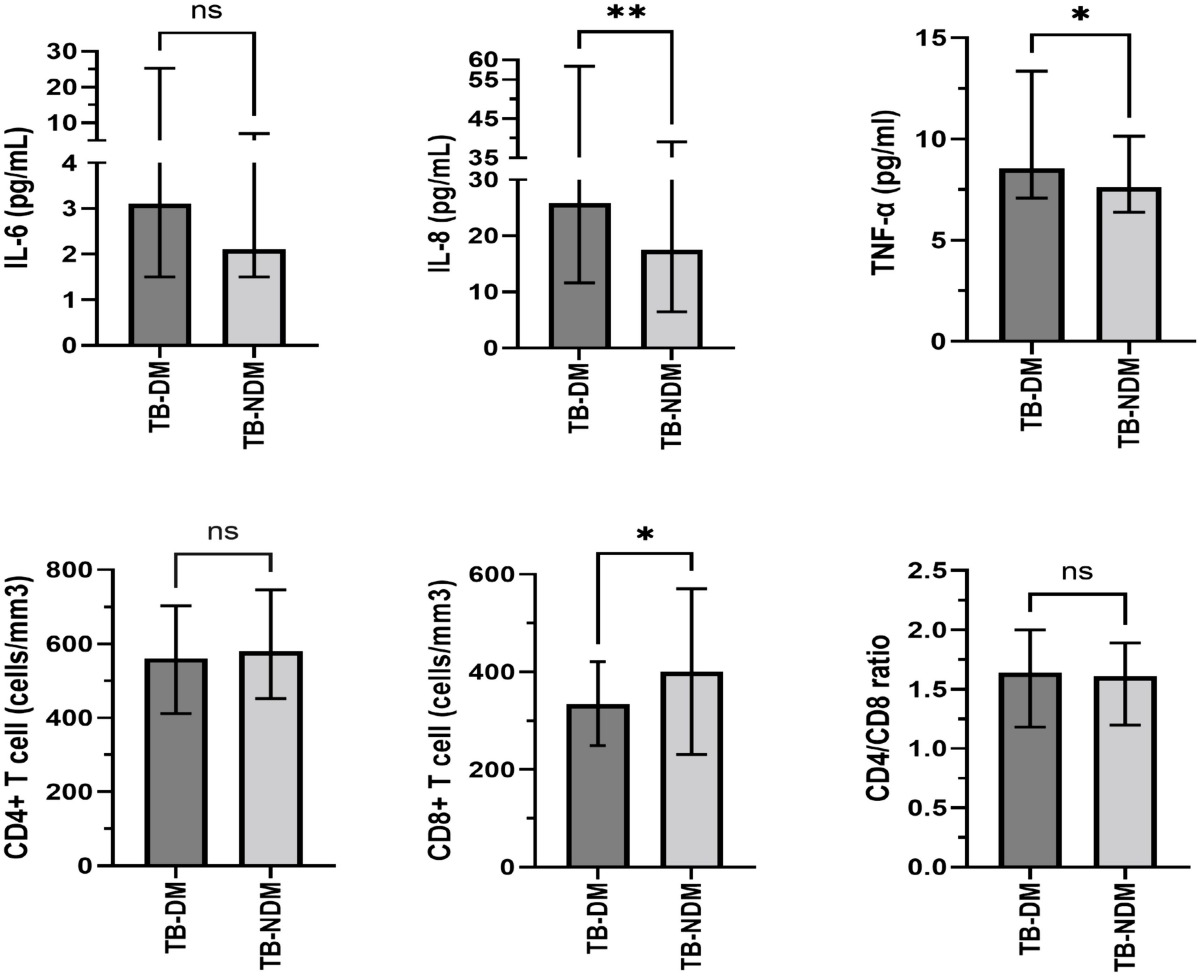
Differences in immunologic features between diabetic and non-diabetic patients. Data was shown median and interquartile range. *p* values were calculated by Mann–Whitney test. *P < 0.05, **P < 0.01.

In present study, inflammatory cytokines such as IL-6, IL-8 and TNF-α were assayed to assess the impact of diabetes on immunological responses (Figure 2). It was found in our study that the median TNF-α in TB-DM group was 8.56 (7.08–13.35) pg./ml, which was significantly higher than that in TB-NDM group [7.64 (6.38–10.14) pg./ml, *p* = 0.033]. Additionally, the levels of serum IL-8 in TB-DM group were also elevated compared with TB-NDM group [25.85 (11.63–58.40 vs. 17.56 (6.44–39.08) pg./ml *p* = 0.003)]. However, there was no significant difference in serum IL-6 concentration between the two groups [3.11 (1.50–25.23) vs. 2.11 (1.50–6.95) pg./ml, *p* = 0.579]. Furthermore, the absolute counts of Lymphocyte subsets in patients with TB-NDM and TB-DM were compared. The absolute counts of CD8+ T lymphocytes are distinctly lower in TB-DM patients [334.02 (249.35–420.71) *VS* 380.95 (291.73–471.25) cells/mm3, *p* = 0.038]. A lower level of CD4+ T lymphocytes were also observed in TB-DM group compared to the TB-NDM group, although differences were not statistically significant (*p* = 0.156). To evaluate the influence of DM control status on immune response, we further analyzed the correlation between DM control status and IL-6, IL-8, TNF-α and so on. There were no differences observed in the levels of IL-6, IL-8, TNF-α, CD4+ T lymphocytes, and CD8+ T lymphocytes between the controlled diabetes group and the uncontrolled diabetes group.

### Treatment efficacy of patients with TB-DM and TB-NDM

All enrolled 160 participants had received standard anti-TB therapy, 39/160 (24.4%) patient had suboptimal treatment response, including 23/80 (28.7%) patients with TB-DM and 16/80 (20%) TB patients without DM. The factors associated suboptimal response rate (SRR) of all patients were analyzed. In univariate analysis, the number of involved lung fields, retreatment for TB, sputum smear positivity, lung cavitary lesions, the levels of IL-8, ESR, CRP and the percentage of CD8+ T lymphocytes were positively related to SRR. IL-6 [OR: 1.02 (1.00–1.04), *p* = 0.099], TNF-α [OR:1.03 (1.00–1.07), *p* = 0.096] and CD4/CD8 ratio [0.59 (0.34–1.04), *p* = 0.065] were marginally associated with SRR. Although the SRR was higher in TB-DM group, there were no statistically significant correlations between DM and SRR. Multivariate logistic regression analysis indicated that retreatment for TB [AOR: 5.68 (2.01–16.08), *p* = 0.001], sputum smear positivity [AOR: 8.01 (2.62–24.50), p = 0.001] were independent prognostic factors for SRR. Furthermore, the factors associated with SRR in TB-DM group were analyzed. There was no clear association between SRR and being overweight or having uncontrolled DM in TB-DM patients. However, it was found that the number of involved lung fields [OR: 1.98 (1.41–2.80), *p* = 0.001], retreatment for TB [OR: 5.83 (2.04–16.70), p = 0.001], sputum smear positivity [OR: 37.72 (4.73–300.38), p = 0.001], lung cavitary lesions [OR: 14.43 (3.09–67.53), p = 0.001], were factors obviously associated with SRR in the univariate model, while multivariate logistic regression analysis revealed that only a sputum smear positivity [AOR: 16.47 (1.75–155.12), *p* = 0.014] was related to the suboptimal treatment response (Table 2).

**Table 2:** Risk factors for suboptimal response rate in TB patients.

	All patients (*N* = 160)				TB patients with DM (*N* = 80)			
Factor	Univariate		Multivariate		Univariate		Multivariate	
	OR (95%CI)	*P* value	AOR (95%CI)	*P* value	OR (95%CI)	*P* value	AOR (95%CI)	*P* value
**Age (year)**	1.01 (0.97–1.05)	0.584			1.02 (0.97–1.07)	0.496		
**Gender**								
Female	0.67 (0.14–3.26)	0.622			0.99 (0.18–5.51)	0.991		
Male	1				1			
**Involved lung fields**	1.49 (1.20–1.85)	0.001	0.99 (0.72–1.37)	0.991	1.98 (1.41–2.80)	0.001	1.63 (0.92–2.91)	0.096
**Retreatment**								
Yes	4.78 (2.22–10.26)	0.001	5.68 (2.01–16.08)	0.001	5.83 (2.04–16.70)	0.001	4.00 (0.90–17.81)	0.069
No	1		1		1			
**Sputum smear positivity**								
Yes	9.99 (4.05–24.64)	0.001	8.01 (2.62–24.50)	0.001	37.72 (4.73–300.38)	0.001	16.47 (1.75–155.12)	0.014
No	1		1		1			
**lung cavitary lesions**								
Yes	5.43 (2.37–12.47)	0.001	1.69 (0.54–5.27)	0.367	14.43 (3.09–67.53)	0.001	1.47 (0.19–11.42)	0.713
No	1		1		1			
**DM**								
Yes	1.61 (0.78–3.35)	0.199						
No	1							
**Overweight**								
Yes	1.69 (0.66–4.33)	0.271			1.83 (0.61–5.52)	0.284		
No	1				1			
**HbA1c ≥ 7.0%**								
Yes					0.61 (0.20–1.82)	0.375		
No					1			
IL-6 (pg/ml)	1.02 (1.00–1.04)	0.099	0.99 (0.96–1.02)	0.631	1.01 (0.99–1.03)	0.477		
IL-8 (pg/ml)	1.02 (1.01–1.07)	0.014	1.00 (0.99–1.01)	0.307	1.003 (1.00–1.007)	0.032	1.002 (0.996–1.008)	0.548
TNF-α (pg/ml)	1.03 (1.00–1.07)	0.096	1.01 (0.99–1.01)	0.859	1.03 (0.99–1.07)	0.205		
CD4+ T cell (%)	0.96 (0.92–1.00)	0.068	1.01 (0.95–1.07)	0.148	0.97 (0.92–1.02)	0.195		
CD8+ T cell (%)	1.06 (1.02–1.11)	0.007	0.94 (0.87–1.02)	0.154	1.07 (1.01–1.13)	0.019	1.10 (0.98–1.23)	0.095
CD4/CD8 ratio	0.59 (0.34–1.04)	0.065	1.08 (0.97–1.20)	0.294	0.64 (0.32–1.28)	0.207		
ESR (mm/h)	1.03 (1.01–1.04)	0.001	1.00 (0.97–1.04)	0.851	1.03 (1.01–1.05)	0.005	1.01 (0.98–1.05)	0.464
CRP (mg/L)	1.01 (1.01–1.02)	0.024	1.01 (0.99–1.03)	0.439	1.01 (0.99–1.02)	0.151		

OR, odds ratio; AOR, adjusted odds ratio; TB, tuberculosis; DM, diabetes mellitus; TNF-α, tumor necrosis factor-α; IL, interleukin; ESR, erythrocyte sedimentation rate; CRP, C reactive protein.

## Discussion

Once MTB enters the body, it initiates inherent and adaptive immunity for resistance. A large number of cytokines participate in immune response, which is closely related to the occurrence and development of disease (15). DM is most notably characterized by disorders of glucose metabolism that may induce alteration in components of the immune system including the change in the levels of specific cytokines and chemokines, altered number and activation state of various immune cell subsets (16). Immunological impairment in DM patients results in heightened susceptibility to tuberculosis. Additionally, the chronic inflammatory environment caused by DM plays a vital role in the increased severity and poor clinical outcome of TB (10).

In this study, despite balancing age, gender, and involved lung fields, there were still several differences in the baseline characteristics of patients with TB-DM and TB-NDM. It was observed that the probability of sputum smear positivity and lung cavitation in patients with TB-DM was higher than that in patients with TB-NDM. Previous researches have demonstrated that the comorbidity of TB and DM promotes the formation of lung cavities (17). In addition, Wei et al. revealed that the number of pulmonary cavities in the TB-DM hyperglycemia subgroup was higher than in the TB-DM euglycemia subgroup (12), suggesting hyperglycemia is a potential risk factor for cavity formation in patients with TB. We also observed that the levels of WBC, CRP, ESR were higher in the patients with TB-DM, which seems to be consistent with precedent studies (18, 19). It was indicated that over-expression of CRP in TB-DM patients was associated with an increased recruitment and infiltration of neutrophils, which led to more inflammation and necrosis (19). ESR also proved to be a sensitive marker for inflammatory response in TB patients and an increased ESR was closely related to the disease activity of TB (20).

Our data showed that TB patients with and without DM displayed different immune responses. It was revealed that the levels of IL-8 and TNF-α in the individuals with TB-DM were significantly higher than those TB patients without DM. Furthermore, the concentration of IL-6 in the serum, although not statistically significant, tended to increase in TB-DM group, suggesting a stronger immune inflammatory response TB-DM patients. TNF-α, IL-6 and IL-8 were once considered as key mediators in protective immune responses for MTB infection (21, 22). Previous studies demonstrated that TNF-α played a crucial role in granuloma formation and maintenance, and thus control of tubercular infection (21). In addition, IL-6, regulated by macrophages was proved to inhibit type I interferon signaling and, consequently, disease progression in the mouse infection models of TB (22). It was reported that IL-8 was released by the macrophages after MTB infection, which will activate and recruit neutrophils to the site of infection and ultimately phagocytose, and kill MTB (23). Recent evidence suggests that IL-6, IL-8, and TNF-α are not only protective in tuberculosis but that the picture is more complex. A growing number of studies correlate the levels of type 1 proinflammatory cytokines (IFN-γ, TNF-α), IL-6 and IL-8 post-infection with enhanced disease burden and poor patient outcome (10, 24), which was in line with the finding in our research. In the present study, correlation analysis reveals an association between IL-6, IL-8, TNF-α and poor treatment response. Antimicrobial and anti-inflammatory combination therapy may be a feasible strategy for patients with TB-DM.

CD4+ and CD8 T+ lymphocytes were thought to play an essential role in the containment of MTB infection (25). It was found in our study that CD4+ T lymphocytes and CD8+ T lymphocytes were lower in the TB-DM group, which was consistent with those of other studies (12). Indeed, recent studies found that compared to TB-NDM patients, the characteristics of patients with TB-DM were an increased frequency of central memory CD4+ and CD8 T+ lymphocytes and a decreased percentage of effector CD4+ and CD8 T+ lymphocytes (9), suggesting that DM changes the distribution frequency of T lymphocyte subsets in patients with TB. In addition, the metabolic homeostasis and function of immune cells are maintained by hypoglycemic therapy during the chronic stages of active TB disease, thus promoting natural host resistance to MTB infection (26). In present study, the count of CD4+ and CD8 T+ lymphocytes appeared independent of suboptimal treatment response, which may be attributed to several confounding factors including blood sugar status and blood sugar control of patients (4), and warrants further investigation.

The presence of DM is associated with delayed culture conversion and poor treatment outcomes in patients with tuberculosis (27). Although in this study, DM was not a factor for increased SRR in the final multivariate analysis, our data indicated that SRR in TB-DM patients was generally higher than those TB patients without DM. The SRR in the present study was close to the previously reported in other studies (6). Univariate and multivariate analysis suggested that retreatment for TB and sputum smear positivity were independent risk factors of poor treatment outcomes in our study. It has revealed that previous TB treatment is an important risk factor responsible for drug resistance and poor TB treatment outcomes (28, 29). In our study, the major cause of retreatment for TB is drug cessation without medical order and insufficient course of treatment. Therefore, it is necessary to strengthen the education and supervision of tuberculosis patients, especially for the migrant population and people with low education levels. Yoon et al. have found initial smear positivity was an independent risk factor for positive culture at 2 months of treatment, which is in accordance with our findings (6). Notably, in the TB-DM group, smear positivity was the only factor associated with increased SRR with a higher odds ratio than that in the overall population. Furthermore, our data showed that TB-DM patients displayed a higher probability of lung cavity formation and positive sputum smear, indicating the potential association between DM and poor treatment response. To enhance treatment response, for the sputum smear positivity patients especially those individuals with comorbid diabetes, it is imperative to strengthen drug-resistant TB detection and optimize the treatment plan.

Inevitably, there were several limitations to this paper. Firstly, due to the retrospective study design, our study is limited in its ability to establish causality between the observed variables. The associations identified between DM and PTB outcomes are correlational, and further prospective studies are necessary to validate these findings and explore causal relationships. In addition, the generalizability of our findings might be limited by the specific demographic and clinical characteristics of the patient population studied, which was confined to a single center in China. Third, this study is based on a small sample size and a short observation period. Several potential confounders (such as glucose level) were not analyzed, which may have potential implications on the result and conclusion of our study.

In summary, our study provides additional evidence for an adverse effect of DM on TB disease severity as reflected by higher occurrence of pulmonary cavity formation, increased proinflammatory cytokine and decreased CD8+ T lymphocytes. Additionally, the data presented here provide further evidence that DM is a potential risk factor for poor treatment response, suggesting that early screening for DM in TB patients should be strengthened. In addition, logistics regression analysis indicated that suboptimal treatment response was associated with retreatment for TB and initial smear positivity in the overall population, whereas in TB-DM patients, only initial smear positivity was found to be an independent risk factor for the occurrence of suboptimal treatment response. Considering the serious threat of TB to the public health, our study suggests the need for appropriate patient education and regular glucose monitoring during the management of TB patients.

## Data availability statement

The raw data supporting the conclusions of this article will be made available by the authors, without undue reservation.

## Ethics statement

The studies involving humans were approved by the Ethics Committee of the Qingchun Hospital, Zhejiang province. The studies were conducted in accordance with the local legislation and institutional requirements. The ethics committee/institutional review board waived the requirement of written informed consent for participation from the participants or the participants’ legal guardians/next of kin because this was a retrospective observational study, and all data were analyzed anonymously, an exemption to patient informed consent was granted by hospitals Institutional Review Board.

## Author contributions

FT: Conceptualization, Data curation, Formal analysis, Methodology, Writing – original draft. JL: Data curation, Formal analysis, Methodology, Writing – original draft. ZL: Data curation, Formal analysis, Methodology, Writing – original draft. ZB: Data curation, Formal analysis, Methodology, Writing – original draft. JC: Conceptualization, Writing – review & editing.
