# Supplementary material for: Clinical features, immunologic parameter and treatment outcome of Chinese tuberculosis patients with or without DM
Source: Front Med (Lausanne). 2024 Jun 12;11:1386124. doi: 10.3389/fmed.2024.1386124 (PMC11199526; doi:10.3389/fmed.2024.1386124)
Supplement: Supplementary file 1 [file Table_1.DOCX]

| Parameter | TB-DM (N=82) | TB-NDM (N=182) |  | TB (N=80) | TB-NDM (N=80) | P Value |
| --- | --- | --- | --- | --- | --- | --- |
| Age [year (IQR)] | 47 (40, 51) | 39 (31, 46) | **0.001** | 47 (39, 50) | 46 (39, 53) | 0.737 |
| Male [n (%)] | 73 (91.5) | 171 (94.0) | 0.441 | 73 (91.3) | 76 (95.0) | 0.534 |
| Involved lung fields [n (IQR)] | 3 (2, 5) | 2 (2, 4) | **0.014** | 3 (2, 5) | 3 (2, 4) | 0.818 |
| Involved lung fields≥3 [n (%)] | 49 (59.8) | 78 (42.9) | **0.012** | 33 (41.3) | 35 (43.8) | 0.873 |
| Retreatment for TB [n (%)] | 26 (31.7) | 44 (24.2) | 0.229 | 26 (32.5) | 25 (31.3) | 0.999 |
| Sputum smear positivity [n (%)] | 44 (53.7) | 43 (23.6) | **0.001** | 43 (53.8) | 27 (33.8) | **0.017** |
| lung cavitary lesions [n (%)] | 46 (56.1) | 63 (34.6) | **0.001** | 45 (56.3) | 31 (38.8) | **0.039** |
| WBC [×109/L (IQR)] | 6.20 (4.98, 7.43) | 5.90 (4.89, 6.90) | **0.047** | 6.20 (5.03, 7.48) | 5.70 (4.80，6.78） | 0.110 |
| CRP [mg/L (IQR)] | 11.15 (2.15, 44.38) | 3.60 (1.00, 12.43) | **0.001** | 11.30 (2.28, 45.73) | 4.00 (1.60, 18.08) | **0.002** |
| ESR [mm/h (IQR)] | 18.55 (10.00, 45.88) | 11.00 (5.00, 20.00) | **0.001** | 18.56 (10.00 46.63) | 12.00 (6.00, 20.23) | **0.001** |

### Table S1 The demographic characteristics of TB patients with and without DM

TB: Tuberculosis; DM: Diabetes mellitus; WBC: White blood cell count; CRP: C reactive protein; ESR: Erythrocyte sedimentation rate; IQR: interquartile range
